# Plasmin and Plasminogen System in the Tumor Microenvironment: Implications for Cancer Diagnosis, Prognosis, and Therapy

**DOI:** 10.3390/cancers13081838

**Published:** 2021-04-12

**Authors:** Alamelu G. Bharadwaj, Ryan W. Holloway, Victoria A. Miller, David M. Waisman

**Affiliations:** 1 Department of Pathology, Dalhousie University, Halifax, NS B3H 4R2, Canada; Alamelu.Bharadwaj@dal.ca (A.G.B.); Ryan.Holloway@dal.ca (R.W.H.); Victoria.Miller@dal.ca (V.A.M.); 2 Department of Biochemistry & Molecular Biology, Dalhousie University, Halifax, NS B3H 4R2, Canada

**Keywords:** plasmin, plasminogen, S100A10, macrophages, tumor microenvironment, uPA, uPAR, PAI-1, PAI-2, metastasis, tumor growth

## Abstract

**Simple Summary:**

In this review, we present a detailed discussion of how the plasminogen-activation system is utilized by tumor cells in their unrelenting attack on the tissues surrounding them. Plasmin is an enzyme which is responsible for digesting several proteins that hold the tissues surrounding solid tumors together. In this process tumor cells utilize the activity of plasmin to digest tissue barriers in order to leave the tumour site and spread to other parts of the body. We specifically focus on the role of plasminogen receptor—p11 which is an important regulatory protein that facilitates the conversion of plasminogen to plasmin and by this means promotes the attack by the tumour cells on their surrounding tissues.

**Abstract:**

The tumor microenvironment (TME) is now being widely accepted as the key contributor to a range of processes involved in cancer progression from tumor growth to metastasis and chemoresistance. The extracellular matrix (ECM) and the proteases that mediate the remodeling of the ECM form an integral part of the TME. Plasmin is a broad-spectrum, highly potent, serine protease whose activation from its precursor plasminogen is tightly regulated by the activators (uPA, uPAR, and tPA), the inhibitors (PAI-1, PAI-2), and plasminogen receptors. Collectively, this system is called the plasminogen activation system. The expression of the components of the plasminogen activation system by malignant cells and the surrounding stromal cells modulates the TME resulting in sustained cancer progression signals. In this review, we provide a detailed discussion of the roles of plasminogen activation system in tumor growth, invasion, metastasis, and chemoresistance with specific emphasis on their role in the TME. We particularly review the recent highlights of the plasminogen receptor S100A10 (p11), which is a pivotal component of the plasminogen activation system.

## 1. Introduction

A tumor is a heterogenous multicellular organ comprised of not only cancer cells but also supporting stromal cells. The environment surrounding the tumor mass is known as the tumor microenvironment (TME). TME is composed of stromal cells that are of diverse types, including fibroblasts, adipocytes, infiltrating lymphocytes and macrophages, lymphatic and vascular network, and most importantly the extracellular matrix (ECM). Moreover, the TME is also composed of secreted proteins, RNAs, exosomes, and microvesicles. The TME is now viewed as one of the hallmarks of cancer because it plays an instrumental role in tumor growth, progression, and metastasis; mediates immune escape; and promotes local therapeutic resistance. The ECM is composed of a diverse array of proteins that are constantly synthesized and remodeled and provide biochemical and biophysical functionality to the TME, which further modulate tumor cell behavior (reviewed in [1,2]). The dynamic remodeling of the ECM and TME is mediated by proteases, such as matrix metalloproteases (MMPs) (reviewed in [3]) cathepsins, and plasmin [4]. Fibrinogen and fibrin are important components of the ECM. Enhanced plasminogen activation is not only associated with increased invasive and metastatic potential of tumor cells but also contributes to tumor growth by activating growth factors and cytokines. In this review, we will discuss the individual components of the plasmin activation system (PAS) and their contribution toward modulating the TME. 

## 2. Proteases in the Tumor Microenvironment

The TME is composed of the ECM and surrounding stromal cells, such as fibroblasts and adipocytes; and infiltrating immune cells, such as macrophages, lymphocytes; pericytes; and endothelial cells [5,6]. The ECM is composed of a network of proteins, such as collagen, elastins, laminin, fibronectin, and proteoglycans, each interacting with one other to form tissue support and architecture [7]. A specialized component of the ECM, the basement membrane (BM), forms the acellular layer that serves as a platform for epithelial and endothelial cells and surrounds fat cells, muscle cells and peripheral nerves. The BM also plays important roles in compartmentalization within tissues and maintenance of epithelial integrity and is a regulator of cell differentiation, proliferation, migration, and survival. The BM contains a variety of proteins including type IV collagen, entactin, laminin, and sulfated proteoglycans. The ECM not only provides biochemical signals for tumor growth, invasion, and metastasis, but it equally contributes to biomechanical properties, such as stiffness, which also influence cellular behavior and phenotype [8,9]. Tumor growth and metastasis not only depend on autocrine signals from tumor cells, but they largely depend on many converging paracrine signals elicited by the host microenvironment and ECM. 

Fibrinogen is a soluble protein produced by hepatocytes and secreted into plasma, where it functions in hemostasis. Thrombin released at the site of vascular injury converts fibrinogen to the insoluble molecule fibrin that forms the major constituent of the blood clot. In 1878, Billroth had reported that tumor cells surround themselves with a fibrin thrombus. Based on this observation, he proposed that fibrin promotes tumor growth and invasion [10]. More recent studies have supported and extended this concept, showing that many hemostatic factors, including fibrinogen, fibrin, and components of the fibrinolytic system, are involved in cancer growth and metastasis [11,12,13]. Fibrinogen but not fibrin is abundant throughout the connective tissue in breast cancer but not in nonmalignant tissues [14]. In contrast, in many other forms of cancer, both fibrin and fibrinogen are present in the tumor stroma [14,15,16,17]. Meta-analysis of studies showed that elevated plasma fibrinogen levels show poor clinical outcome in solid tumors and hematological cancers, though the results for hematological cancers were contradictory [18,19]. It appears that fibrin promotes cell migration by providing a matrix for tumor cell migration and by interacting with adhesive molecules of the cancer cells. Fibrinogen can serve as a scaffold that binds tumor cells and platelets as they leave the tumor site. As an extracellular protein matrix, fibrinogen not only promotes the migration of tumor cells but also protects tumor cells from the innate immune surveillance system [20]. However, it also presents a barrier that must be breached before extravasation into the blood can occur. Therefore, proteolysis of fibrin must be carefully regulated as excessive fibrinolysis will prevent its utilization for the migration of the cancer cells and insufficient fibrinolysis will not allow the cancer cells to pass through the fibrin barrier [21,22,23]. 

Enhanced protease expression and proteolytic activity are hallmarks of cancer progression [24,25]. These proteases participate in the invasion and metastasis of tumor cells by promoting the degradation of the ECM. However, it has been recently suggested that their role is much more complicated, and they participate in multiple stages of cancer progression. Extensive studies have demonstrated that proteolytic activity is elicited not only by tumor cells but also by stromal cells; this concerted proteolytic activity is responsible for ECM degradation and remodeling in a TME [26]. There are five classes of proteases that are categorized based on their mechanism of activity and active site residue, namely aspartic, cysteine, metallo, serine, and threonine proteases [26,27,28,29,30]. The activity of these proteases is tightly regulated by the presence of protease inhibitors which are classified based on the proteases they inhibit. Cystatins inhibit cysteine proteases; serpines (serine protease inhibitors) inhibit serine proteases; and tissue inhibitors inhibit metalloproteases. The threonine proteases are inhibited by bortezomib [31,32]. Further regulation of proteolytic activity is mediated by gene expression and epigenetic regulation, localization and compartmentalization, trafficking, post-translational modification, and zymogen activation [33]. Although this review focuses on the plasminogen/plasmin system it is important to point out that the other classes of proteases also play an important role in the disease process. For example, overexpression of the cysteine protease, cathepsin B has a well-established link to tumor growth, invasion, angiogenesis and metastasis [34,35]. The threonine protease activity of the proteasome plays a key role in protein degradation and its deregulation has been linked to malignant progression [36]. The caspases, are a family of cysteine-dependent aspartate-specific proteases that have an important function in the maintenance of cellular and organismal homoeostasis by functioning as mediators of the inflammatory response and apoptosis [37]. The topic of the role of the different classes of proteases in cancer has been reviewed in [38] and will not be the focus of detailed discussion in this review. 

## 3. Overview of the Plasmin–Plasminogen Activation System

The first publication by Virchow on the decomposition of fibrin in the blood of dissected human bodies appeared in 1846. The term fibrinolysis was introduced in 1893 by Dastre who published observations on the dissolution of clotted dog’s blood [39]. Fibrinolysis is the process of degradation of fibrin, which is the major component of a thrombus [40,41]. The process involves interactions between multiple components of the fibrinolytic system, which play a major role in the dissolution of clots during wound healing and restoration of circulation. The fibrinolytic system is involved not only in homeostasis but also in the progression of several pathological conditions, such as cardiovascular diseases; angiogenesis; and tumor invasion and metastasis [42]. In fact, extracellular matrix (ECM) degradation is one of the most crucial steps involved in local invasion and distant metastasis [1]. The critical mediator of fibrinolysis is plasmin—a potent serine protease. Plasmin also plays a key role in ECM degradation directly and indirectly by activating many of the proteases that function in matrix degradation. Plasmin is generated from its precursor zymogen, plasminogen, by the cleavage of the Arg561–Val562 peptide bond by tissue plasminogen activator (tPA) and urokinase-type plasminogen activator (uPA) [43]. A number of key papers from MacFarlane in 1946 and Astrup in identified the presence of plasminogen activators and regulators [44,45].

**Figure 1:**
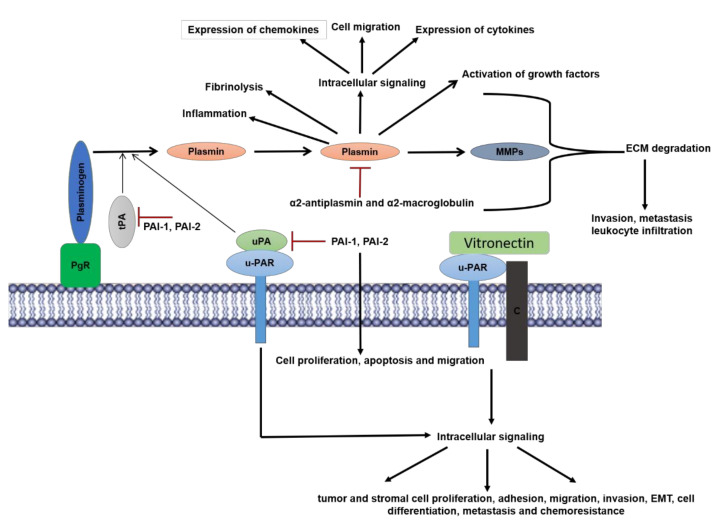
Overview of components and functions of plasminogen-activation system: Plasminogen is activated to the serine protease plasmin by a multicomponent system comprised of plasminogen receptors (PgR), urokinase-type-plasminogen activator (uPA), and tissue-plasminogen activator (tPA). uPA is bound to its receptor urokinase-plasminogen activator receptor (uPAR) whereas in most cases tPA is bound by the PgR. The activity of tPA and uPA are inhibited by the plasminogen-activator-inhibitor 1, 2 (PAI-1, PAI-2), of which PAI-1 is a more potent inhibitor. PAI-1 also promotes cellular proliferation, apoptosis, and migration. Plasmin activity is directly regulated by inhibitors α2 anti-plasmin and α2-macroglobulin. Plasmin mediates its physiological effects by virtue of its protease dependent and independent activities. Plasmin proteolytically activates other proteases such as matrix metalloproteinases (MMPs) that along with plasmin is involved in the degradation of extracellular matrix proteins (ECM) which promotes inflammation including leukocyte infiltration in tumors, cancer cell invasion and metastasis. Plasmin promotes tumor growth by proteolytically activating nascent growth factors in the ECM. Intracellular signaling by plasmin via several receptors (described above) mediates a plethora of effects that include expression of cytokines and chemokines, cell migration and inflammation. uPA and uPAR function both in plasminogen-dependent (protease activity) and plasminogen-independent mechanisms that promote multiple cellular responses. uPAR functions independently of uPA by binding to vitronectin and co-receptors (C) such as integrins, to promote intracellular signaling, which result in tumor and stromal cell proliferation, adhesion, migration, invasion, epithelial-mesenchymal-transition (EMT), cell differentiation, metastasis and chemoresistance in cancer cells.

Plasminogen is synthesized and secreted by the liver [46]. In processes, such as wound healing and tissue remodeling that occurs in the TME, uPA is the predominant form of plasminogen activator, whereas tPA is the primary activator in the circulation [47]. While tPA is found in the ECM of most tissues, uPA is localized to cell surfaces by its receptor uPAR. The activities of both tPA and uPA are regulated by plasminogen activator inhibitor (PAI)-1 [48] and PAI-2 [49], whereas the activity of plasmin itself is inhibited by α2-antiplasmin [50,51] and α2-macroglobulin [52] (Figure 1). Overall, a complicated interplay between the plasminogen activators and inhibitors determines the extent of tissue remodeling, fibrinolysis, and tumor invasion and metastasis [53,54]. In the TME, this is further complicated by the fact that the expression of the activators and inhibitors are not restricted to tumor cells alone, but the stromal cells are also involved in the expression and secretion of activators and inhibitors. 

Plasmin generation proceeds at a slow rate but is accelerated by a group of cell surface proteins that bind plasminogen, called plasminogen receptors [55,56,57]. Plasminogen receptors localize the bound plasminogen near the vicinity of the tumor cell, allowing easy access by uPAR-bound uPA. Plasminogen receptors are also present on the cells of the TME, including endothelial cells, activated myofibroblasts, and macrophages. Thus, there is substantial interaction between the tumor and host cells for plasminogen binding, plasmin generation, and signaling crosstalk between the two components. These interactions play a vital role in cancer progression by modulating cell proliferation, angiogenesis, invasion, and metastasis. 

## 4. Structure and Function of Plasminogen and Plasmin 

Plasminogen is a 92–94 kilodalton protein that circulates as a zymogen in the blood. The variation in molecular weight is due to glycosylation differences, discussed further below. Plasminogen, like many hemostatic proteins, is produced in the liver and is found at a concentration of approximately 2 µM in the plasma [58]. It is a multidomain protein, comprised of an amino (N)-terminal peptide domain, five kringle domains, and the serine protease domain [59]. The classical catalytic triad is located within the serine protease domain: Histidine 603, Aspartate 646, and Serine 741. This domain has a homology with the active site of other serine proteases, such as trypsin and elastase [60]. The kringle domains of plasminogen are so-called due to their physical resemblance to kringle pastries [61]. The 3-dimensional structure of the kringle domains revealed that multiple intra-molecular disulfide bonds contribute to the structural rigidity of these domains [62]. The kringle domains contain sites that bind lysine residues. The lysine-binding sites (LBS) of plasminogen kringles bind lysine residues both within the plasminogen molecule and those contributed by fibrin blood clots and cell-surface plasminogen receptors [60]. The crystal structure of plasminogen has been solved and revealed much about the architecture of the molecule [63,64]. The LBS of all kringle (Kr)domains, except Kr are engaged in intramolecular interactions, while only Kr1-LBS is readily available for extra molecular binding. Plasminogen circulates in a “closed conformation,” with Lys50, Arg68, and Arg70 from the N-terminal peptide domain interacting with the LBS of Kr4 and Kr5, respectively, and Lys708 interacting with the LBS of Kr2. In contrast, the LBS of Kr1 and Kr4 have an LBS that binds with higher affinity to the C-terminal lysines of fibrin structures. These interactions between the N-terminal domain and intramolecular residues are critical for maintaining plasminogen in a tight closed conformation. The proteolytic cleavage at Lys77 by plasmin results in the removal of the N-terminal region, which results in the formation of a truncated form of plasminogen called Lys-plasminogen [65,66,67]. This form of plasminogen is in a more open conformation. Law [64] had proposed that a plasminogen receptor likely interacted with both Kr1 and Kr5. The interaction with Kr1 was responsible for the tethering of the molecule to the plasminogen receptor, whereas the subsequent interaction with Kr5 unleashed the series of conformational changes responsible for the exposure of the activation loop to cleavage by plasminogen activators.

Both, the rate, and location of plasmin formation, are tightly controlled. To ensure hemostasis, plasmin generation is typically restricted to the sites of thrombosis and angiogenesis and the sites of ECM remodeling. Unrestrained plasmin generation is a factor in the depletion of pro-coagulant proteins in disseminated intravascular coagulation [68]. Plasmin generation on the surface of tumor cells leads to cell invasion and metastasis [69]. Local surfaces play a part in plasmin generation; both the fibrin surface and cell surface carry out a similar regulatory role [70]. Plasminogen receptors found on the cell surface, some of which also bind tPA, colocalize with other receptors that specifically bind plasminogen activators [71,72,73,74]. The plasminogen receptors also provide lysine residues for binding plasminogen and in certain cases, a site for binding tPA. As has been shown for p11, the binding of tPA and plasminogen/plasmin to this receptor protects tPA from inactivation by PAI-1 and plasmin from inactivation by alpha-2 anti-plasmin [75]. Similarly, on the fibrin clot surface, C-terminal lysine residues are interspersed within the fibrin network. Restricting plasmin activation to either a cell surface or the clot surface allows for proteolytic targeting as well as some protection from circulating inhibitors. In addition to its cleavage to form plasmin, plasminogen can also be proteolysed into several bioactive molecules called angiostatins [76]. The angiostatins are a group of proteolytic products derived from plasminogen, which retain some of the kringle domains but lack the proteolytic domain. Structures include Kr1-Kr3, Kr1-Kr4, and Kr1-Kr5 [77]. Our laboratory demonstrated that the autoproteolytic cleavage of the Lys468–Gly469 bond and reduction of the plasmin Cys462–Cys541 disulfide of plasmin resulted in the formation of an angiostatin, A61 (Lys78–Lys468) [78]. The angiostatins are crucial inhibitors of vascular endothelial cell proliferation, tube formation, and migration; they can also promote vascular endothelial cell apoptosis [79]. 

Plasmin has proteolytic activity against a broad range of substrates, such as syndecans [80], laminins, fibronectin [81], vascular cell adhesion protein 1 [82], and vitronectin [83]. The broad range of substrates results in an extensive range of processes, both physiologic and pathologic, in which plasmin participates. These processes include fibrinolysis, signaling pathways, cell migration, wound healing, inflammation, and oncogenesis [84]. Plasmin releases growth factors such as fibroblast growth factor-2 (FGF-2), hepatocyte growth factor (HGF), and vascular endothelial growth factor (VEGF) that are sequestered by the ECM [85,86,87,88]. Additionally, plasmin activates MMPs, such as MMP 1, 2, 3, 9, and 14, and indirectly affects tumor cell invasion [89]. Plasmin also promotes intracellular signaling through various pathways to promote cell migration of peripheral blood monocytes through Protease-activated-receptor 1 (PAR-1) [90,91]. Stimulation of monocytes by plasmin results in enhanced expression of inflammatory cytokines (tumor necrosis factor-alpha (TNFα), interleukin (IL)-6, IL-1α/β, CD40) and chemokines (monocyte chemoattractant protein (MCP)-1/CCL2), tissue factors, and lipid mediators via activation of AP-1 and nuclear factor-kappa (NF-κB) transcription factors [90]. Plasmin has been shown to activate multiple signaling pathways, such as Janus Kinase/Signal Transducer and Activator of Transcription (JAK/STAT), p38 mitogen-activated protein kinase (MAPK), extracellular signal-regulated protein kinase (ERK)1/2 [92]. With HT29-M6 intestinal epithelial cells, Diaz et al. showed that plasmin plays a dual role in promoting cell scattering as a result of its ECM degrading proteolytic activity and in intracellular signaling by the induction of ERK1/2 activation [93]. Some cells, such as Chinese Hamster Ovary and BA3 cells, utilize both the proteolytic activity and the signaling function of plasmin to induce cell migration. Specifically, Tarui et al. showed that plasmin bound to αvβ3 integrin and promoted cell migration in these cells, whereas treatment with plasmin inhibitor aprotinin abrogated signaling and migration [94]. In summary, plasmin activates several cell types, such as monocytes, macrophages, fibroblasts, endothelial cells, epithelial cells, and platelets, that form a part of the TME and promotes their migration. 

## 5. Plasminogen Activators

There are two physiologic activators of plasminogen—uPA and tPA—which are used clinically as thrombolytic therapeutics [40]. Both tPA and uPA are multidomain proteases. The active forms of each of these enzymes are inhibited by PAI-1 and PAI-2. The tPA is a 70 kDa protein of 527 amino acids [95]. It consists of 2 kringle domains, a finger domain, epidermal growth factor (EGF)-like domain, and serine protease domain [96]. The tPA interacts with fibrin via its finger domain as well as kringle-2. Like most of the kringle domains of plasminogen, kringle-2 is a lysine binding site; however, kringle-1 has a serine residue at the site of a usual tryptophan residue within the hydrophobic cleft that alters its ability to bind to lysine residues [97,98]. The tPA is synthesized and secreted by endothelial cells as well as many brain cells, including neurons, astrocytes, microglia, and oligodendrocytes [96,98]. The plasma protein concentration of tPA is in the range of 5–10 µg/L. The protein is synthesized as a proenzyme single chain, cross-linked polypeptide, glycosylated at threonine Thr61 (O-linked), asparagine Asn117, Asn184, and Asn448 (N-linked). The proenzyme has a low serine protease activity. This single-chain form is fully activated by cleavage of the arginine Arg275-isoleucine Ile276 bond by plasmin, kallikrein, or coagulation factor Xa. This 2-chain form is held together by a single disulfide bond between Cys264 and Cys395 [96]. The interaction of tPA with endothelial cells and vascular smooth muscle cells can lead to increased activation of plasminogen. Previous studies have proposed that a C-terminal lysine or arginine residue on annexin A2 (also known as p36), generated by an unknown protease as the binding site for plasminogen [99,100]. Subsequent studies have thrown doubt on the existence of proteolyzed annexin A2 as the binding site; it is more likely to be the binding partner of annexin A2, p11, which possesses a C-terminal lysine residue [100,101]. Like tPA, uPA is found in the plasma at approximately the same concentration of 5–10 µg/L, but it is produced by cells in the lungs, kidneys as well as keratinocytes and endothelial cells. The uPA is synthesized as a 55 kDa single-chain protein consisting of 411 amino acids [102]. This multidomain protein has an EGF-like domain, one kringle domain, and a serine protease domain. Additionally, like tPA, it is glycosylated: Thr18 (O-linked) and Asn302 (N-linked). Additionally, it has 2 phosphorylation sites: serine Ser138 and Ser303. The secreted protein is a zymogen and requires cleavage of its Lys158–Ile159 bond to demonstrate its full activity. The two-chain form is held together by a single disulfide bond between Cys148 and Cys279. The Lys–Ile bond is cut by plasmin, kallikrein, coagulation factor XIIa, or cathepsin B [103,104]. This results in the high molecular weight form of active uPA. Additionally, there is a low molecular weight form, primarily found in the urine and is generated by plasmin or auto-proteolysis at the Lys135–Lys136 bond. The single-chain pro-uPA is 250-fold less potent in the activation of plasminogen than the two-chain uPA. Different proteases have been reported to function in mediating the cleavage of pro-uPA. Plasmin most effectively converts the pro-uPA into active uPA [105]. Among the other proteases that can activate pro-uPA includes cathepsin B and L, nerve growth factor-g, trypsin, kallikrein, thermolysin, and mast cell tryptase [103,104,106,107,108,109]. uPA is primarily responsible for cell surface-associated plasminogen activation. This is accomplished by the binding of the single-chain proenzyme to the glycosylphosphatidylinositol (GPI)-anchored uPAR, where the proenzyme is activated by plasmin, cathepsin B, or kallikrein [54]. The activation of cell-bound plasminogen by receptor-bound uPA is characterized by a 40-fold reduction in the Km for plasminogen activation [74]. The co-localization of uPAR with plasminogen receptors localizes the resultant plasmin activity to the cell surface. The cell membrane-bound plasmin activity created by cell-bound plasminogen is protected by inactivation from plasma-borne inhibitors, but its activity can be indirectly reduced by the PAI-1 and PAI-2 through the inhibition of tPA and uPA [110,111]. In parallel, plasmin bound to the surface of cells cleaves pro-uPA bound to uPAR at a rate 50-fold that of plasmin in solution [41]. This directed activation on the cell surface gives those cells an invasive phenotype. Invasive cells make use of the broad substrate specificity of the resulting plasmin to degrade the various proteins of the ECM, including fibronectin, laminin, vitronectin, and proteoglycans. As discussed, plasmin can also activate pro-MMPs, resulting in the degradation of collagen [54]. 

The coordination of the uPA system consisting of plasmin and uPA along with the inhibitors alpha2-antiplasmin, PAI-1 and PAI-2 together with the receptor uPAR, plays an important role in the regulation of cancer progression and metastasis [54,58]. The first evidence of a possible causative role of plasminogen activator in malignancy was shown by the sharp increase in fibrinolytic activity in virally transformed fibroblasts [112]. This plasminogen activator was later identified to be uPA [113]. Increased concentration of uPA in the plasma is correlative of poor prognosis in breast, bladder, gastric, and prostate cancers; head and neck and colorectal cancers; and melanoma [114]. 

As discussed, uPAR is a GPI-anchored membrane receptor for uPA, that regulates protease activity on the cell surface. Later studies have shown that it binds to vitronectin from the ECM with the aid of co-receptors, such as integrins, caveolin, and G-protein-coupled receptors, and promotes intracellular signaling and gene expression [115,116]. Interestingly, binding of uPA to uPAR also activates intracellular signaling that is mediated by the N-terminal region of uPA and is independent of the active site domain, suggesting a plasmin-independent mechanism [117]. The cell signaling cascade initiated by uPAR promotes cancer cell invasion, migration, epithelial to mesenchymal transition, cell differentiation, proliferation, and metastasis. The expression of uPA and uPAR gene is elevated in many cancers compared to non-cancerous tissues. The uPAR binds to uPA and at the same time binds to vitronectin, which is relevant for the downstream signaling by uPAR. The uPAR promoter is activated by several transcription factors, such as activator protein 1, NF-κB, and hypoxia-inducible factor 1α, which are upregulated by the RAS-ERK-MAPK pathway and TME factors such as hypoxia (reviewed in [118]). The uPAR is also cleaved at its GPI-anchor by a specific phospholipase and is found as a soluble form (suPAR) in the plasma and urine [119]. The suPAR can activate the G-protein coupled chemotactic receptor FPRL1/LXA4R. The expression of uPAR is upregulated by various inflammatory cytokines, such as TNF-α, interferon-gamma (IFN-γ), IL-1, and IL-2 [120]. More recently, it has been demonstrated that uPAR is upregulated during senescence and uPAR specific-chimeric antigen receptor T-cell therapy enhances the survival of mice with lung adenocarcinoma [121]. Expression of uPA and uPAR has been observed in various hematopoietic cells, and both these proteins play an important role in the regulation of innate and adaptive immune response through the modulation of cell adhesion and migration of neutrophils, monocytes, macrophages, and myeloid-derived-suppressor cells and activation of T-cells [114]. Thus, the uPA–uPAR axis potentiates tissue remodeling not only in the cancer cells but also in the immune cells by allowing their invasion, migration, and adhesion in the stroma where they modulate tumor growth and metastasis. The uPA–uPAR axis also regulates apoptosis. When uPA and uPAR were downregulated simultaneously, a dramatic decrease in the invasiveness of breast cancer cells was observed along with the activation of the apoptotic cascade [122]. Furthermore, antibody-targeted inhibition of u-PAR reduced metastasis and induced apoptosis in ovarian cancer cells In Vitro and In Vivo [123].

## 6. Plasminogen Activation Inhibitors

PAI-1 is a 379-amino acid serine protease inhibitor that inhibits the activity of tPA and uPA [124,125,126] which is associated with inflammation and tumorigenesis. Interestingly although PAI-1 functions as an inhibitor of uPA and tPA, it serves a pro-tumorigenic role in various cancers such as lung and breast [127,128,129]. This is clearly because PAI-1 is a multifunctional protein not only regulating plasmin generation but also modulating cell proliferation, migration, and apoptosis (reviewed in [130]). Moreover, PAI-1 plays both an autocrine and paracrine role in tumorigenesis and is produced by both tumor cells and cells of the TME such as adipocytes, macrophages, fibroblasts, smooth muscle cells, and endothelial cells. Much of our knowledge of the pro-tumorigenic and metastatic role of PAI-1 was deciphered from PAI-1-deficient mouse models and transgenic tumor models. Interestingly, the tumor growth and metastasis phenotype in syngeneic models and genetically engineered mouse tumor models provided contradictory results. The implantation of PAI-1 deficient syngeneic tumor cells in PAI-1 knockout (KO) mice demonstrated that both host and tumor cells PAI-1 contributed positively to tumor growth, angiogenesis, and metastasis in a dose-dependent manner [128,131]. However, loss of PAI-1 in the transgenic breast [132] and ocular [133] tumor models did not show any significant effect on tumor development and progression possibly due to the compensation by other members of the PAI family and other protease inhibitors [134]. The function of PAI-1 in tumor angiogenesis, migration/invasion, and apoptosis is mediated by distinct structural domains. For instance, the pro-angiogenic function is due to the vitronectin binding domain and uPA-binding domain (anti-protease). This is primarily by two mechanisms one is plasmin dependent and the other is plasmin independent. First, the inhibition of uPA by PAI-1 binding prevents the activation of plasminogen to plasmin, which further prevents cleavage of Fas-L on the cell surface and consequently, Fas-mediated apoptosis of endothelial cells is inhibited [129]. Second, PAI-1 binding to vitronectin allows the release of endothelial cells from vitronectin and migration for promoting angiogenesis [135]. Finally, the ability of PAI-1 to bind to its receptor low density lipoprotein receptor-related protein 1 (LRP1) promotes intracellular signaling and tumor cell and endothelial cell migration [136]. A recent study has highlighted the mechanistic role of the pro-tumorigenic function of PAI-1 in the TME. This study demonstrated that PAI-1 mediates a pro-tumorigenic effect by recruiting and polarizing macrophages to M2 phenotype in the TME. Interestingly, this dual role is promoted by two different structural domains of PAI-1. The LRP1 domain mediates the monocyte migration and recruitment, and the uPA interacting domain causes the M2 polarization via activation of the IL-6/STAT3 pathway which in turn is induced by the p38-MAPK and NF-κB signaling pathways. Although it is unclear how PAI-1 activates these signaling pathway, the authors speculate that reduced plasmin in the presence of PAI-1 potentially prevents receptor protein degradation and enhances downstream signaling [137]. 

PAI-2, although originally identified as a less potent inhibitor of uPA, is a 47 kDa protein. It has been now shown to having a predominantly intracellular function and has been discovered to be a modulator of proteotoxic stress [138] unrelated to plasminogen activation/uPA inhibition. The loss of PAI-2 gene in mice is not accompanied by any major phenotypic changes associated with fibrinolysis or hemostasis unlike the PAI-1 deficient mice [139,140]. However, bioinformatics analysis and expression data from macrophages have revealed that PAI-2 is modulated with genes associated with cell migration and movement. There are some reports suggesting a role for monocyte-derived PAI-2 in promoting uPA mediated fibrinolysis or clot resolution [141]. 

## 7. Plasminogen Receptors 

Plasminogen binds to cells with low affinity (K_d_ = 0.3–2 µM) and high capacity (10^4^–10^7^ binding sites per cell) [73,142]. The plasminogen-binding sites on cells are mediated by both proteins and non-proteins such as glycosaminoglycans and gangliosides. 

Plasminogen receptors serve as anchoring sites for plasminogen on the cell surface. The binding of plasminogen to these receptors increases the rate of local plasmin generation. Plasminogen receptors are found on the surface of most cell types (except for red blood cells) at a high density, dependent on cell type: 1to 200 × 10^5^/endothelial cell, 5 × 10^5^/lymphocyte, and 35,000/platelet [57,68]. 

The current dogma states that although both internal lysines and the carboxyl-terminal lysine of plasminogen receptors bind to plasminogen, it is the plasminogen receptors with carboxyl-terminal lysine residues that preferentially induce a conformational change in plasminogen that greatly facilitates its activation by the plasminogen activators. This dogma is based on the report that treatment of U937 cells with carboxypeptidase B (CpB) reduced plasminogen binding by about 60% whereas plasminogen activation was reduced by about 95% [72]. Since CpB proteolytically removes lysine and arginine residues from the carboxyl-terminal of proteins, it was concluded that only a subset of plasminogen receptors, that have a carboxyl-terminal lysine residue, participate in plasminogen activation. The analysis of the effect of CpB on plasminogen activation by THP-1 monocytes, THP-1 macrophages, and human umbilical vein endothelial cells revealed that carboxyl-terminal lysines played only a minor role in plasminogen activation [143]. Similarly, CpB treatment of activated platelets reduced plasminogen activation by only about 20% [144]. These studies have suggested that plasminogen receptors with internal lysine residues also participate in plasminogen activation by plasminogen receptors. 

An understanding of the mechanism of interaction of an internal or C-terminal lysine with the LBS of plasminogen comes from structural studies. The interaction of lysine with the LBS is mediated by hydrophobic interaction as well as by charged residues. Lysine is a basic aliphatic amino acid with a side chain consisting of a 4-carbon chain hydrophobic region and a positively charged amine group. The amino group of lysine interacts with negatively charged aspartic acid residues within the binding pocket of the LBS, whereas the hydrophobic chain interacts with the hydrophobic cleft of the LBS [60]. Compared to an internal lysine residue, the carboxyl-terminal lysine possesses a negatively charged carboxyl group which interacts with the cationic center of the LBS of plasminogen. 

Localizing plasmin activity at the cell surface grants the cell various abilities. The broad spectrum of plasmin activity means that in addition to direct extracellular proteolysis, plasmin can also activate other proteinases, thereby increasing the overall proteolytic activity of the cell. Plasmin is also able to process latent growth factors and cytokines in the ECM that induces signaling events to modulate cellular functions such as cell migration. 

Plasminogen receptors are often found co-localized with receptors for plasminogen activators or bind tPA itself [145,146]. The co-localization of plasminogen and its activators aids in restricting the activity of the resulting plasmin to the cell surface.

Many proteins have been identified as potential plasminogen receptors. Most of these proteins have C-terminal lysines. Those that have a C-terminal lysine include α-enolase [142], histone H2B [147,148], S100A4 [149], the annexin A2 heterotetramer (annexin A2 in complex with p11 also known as AIIt) [101], plasminogen (Plg)-RKT [150], TIP49a [151], and cytokeratin 8 [152]. Examples of plasminogen receptors that do not have the classic C-terminal lysine residue include high mobility group box-1 protein (HMGB-1) [56] (also known as amphoterin) and integrins, primarily those of the β2 integrin family [57] and glucose-regulated protein 78 [153]. It has been shown that a significant amount of the plasmin generating activity of malignant cancer cells is due to a novel, unidentified class of plasminogen receptors. These plasminogen receptors are generated by the action of plasmin on unknown cell surface proteins, resulting in the generation of a new carboxyl-terminal lysine and hence a new plasminogen-binding site. For example, the majority of the plasmin-generating capacity of several malignant breast cancer cell lines is due to these unidentified, cryptic plasminogen receptors [154]. 

Many of the plasminogen receptors are associated with cancer and are found on the surface of most tumors [155]. Examples of plasminogen receptors that are upregulated in cancer include p11 (reviewed in [156]), S100A4 [157,158], α-enolase [159], and cytokeratin K8 [152]. Interestingly, the majority of plasminogen receptors function in tumor-stromal cross-talk during cancer progression. The expression of plasminogen receptors, such as p11 and Plg-RKT, in macrophages, promote pro-tumorigenic phenotype in animal models. Importantly, these receptors have been shown to play a predominant role in macrophage recruitment to tumor sites and during inflammation and will be discussed in detail in the later sections. In this review, we will focus on the plasminogen receptor p11 that has contributed greatly to our understanding of plasminogen receptors in the TME and tumor progression. 

## 8. AIIt Structure, Function, and Regulation

The annexin A2-S100A10 heterotetramer, is also known as AIIt [78,101,160]. AIIt is composed of two subunits of annexin A2 (also called p36) and a dimer of p11. The AIIt heterotetramer acts as a platform for promoting interactions with other cellular proteins. It regulates multiple ion channels, such as Cl^−^, K^+^, Na^+^, Ca^2+^, cystic fibrosis transmembrane conductance regulator, transient receptor potential vanilloid type 5 and 6, NaV1.8, Acid-Sensing Ion Channel-1, and TASK1 using distinct mechanisms, which has been reviewed in detail elsewhere and will not be discussed in this review [161]. 

**Figure 2:**
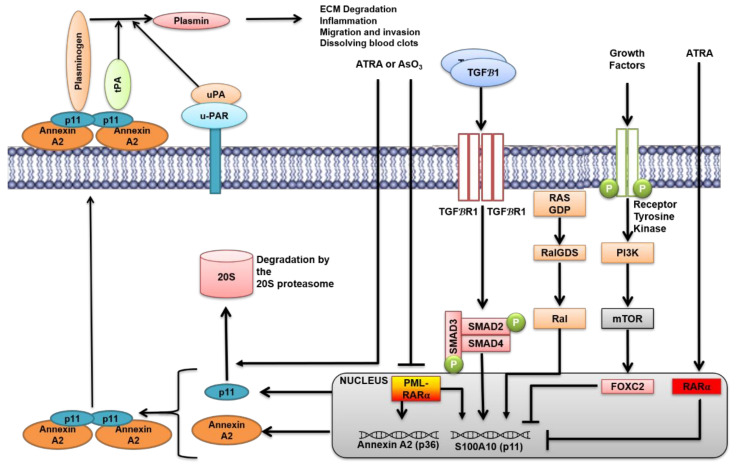
Regulation of S100A10 (p11): P11 is transactivated by (1) the promyelocytic leukemia-retinoic acid receptor alpha (PML-RARα) fusion oncoprotein, (2) TGFβ1-dependent activation of the SMAD pathway, and (3) oncogenic RAS-mediated activation of the Ral-GDS/Ral pathway. Conversely, transcriptional repression of P11 is mediated by (1) ATRA- and arsenic trioxide (AsO3)-induced degradation of the PML-RARα fusion oncoprotein, (2) growth factor-induction of the PI3K/mTOR pathway and consequent FOXC2-dependent transcriptional repression of p11, and (3) ATRA-activation of RARα. Newly transcribed p11 and p36 (Annexin A2) proteins rapidly form the AIIt heterotetramer complex within the cytoplasm prior to being transported to the cell surface. Although the p11–p36 interaction protects p11 from degradation by the 26S proteasome, ATRA and AsO3 both induced the ubiquitin-independent degradation of p11 by the 20S proteasome. Once at the cell surface, AIIt acts as a dual receptor for plasminogen and tissue plasminogen activator (tPA) and co-localizes with and urokinase-type-plasminogen activator/uPAR complex. By localizing plasminogen and its activators, AIIt catalyzes the cleavage of plasminogen to produce plasmin, a serine protease involved in ECM degradation, inflammation, cellular migration an invasion, and blood clot dissolution.

AIIt is also a significant contributor to plasmin generation of many cell types. AIIt binds both tPA and plasminogen on the cell surface and converts plasminogen to plasmin. The p11 subunit (Mr 11 kDa) of AIIt is one of 20 different members of the S100 family made of calcium-binding, dimeric EF hand-type proteins. p11 is present in several tissues, such as the brain, heart, gastrointestinal tract, kidney, liver, lung, spleen, testes, epidermis, aorta, and thymus. It is highly expressed in many cell types, such as endothelial, cardiomyocytes, monocytes, T lymphocytes, and neurons [162]. The p11 subunit of the AIIt heterotetramer is one of the most highly regulated plasminogen receptors. These regulators include physiological signals such as EGF [163,164] and IFN-*γ* [165,166]. p11 is also regulated by oncogenes, such as KRAS [167], which is present in about 30% of all human cancers and promyelocytic leukemia-retinoic acid receptor alpha (PML/RARα) oncoprotein [168], the oncogene responsible for acute promyelocytic leukemia (Figure 2). Our laboratory has shown that p11 is regulated by oncogenic RAS by the Ral-GDS pathway and depletion of p11 in RAS transformed cells results in a substantial reduction in plasmin generation and plasminogen dependent invasion [167] (Figure 2). The expression of p11 is also regulated by glucocorticoids, cytokines, growth factors, and neurotransmitters [162,169]. The expression of p11 is aberrantly regulated in many pathological conditions, such as cancer, depressive mood disorder, and neurodegeneration [170].

The large subunit of AIIt, p36, is a 36-kDa protein belonging to a group of calcium-dependent, phospholipid-binding proteins known as the annexin family [162,171,172]. The formation of the AIIt heterotetramer occurs intracellularly when the p11 homodimer becomes attached to two copies of a p36 subunit. Within the heterotetramer, p36 has two key functions: (1) to facilitate the localization of p11 to the cell surface, [173] and (2) to prevent the rapid degradation of newly translated p11 since the binding of p36 and p11 blocks p11 from ubiquitylation and degradation [174,175,176]. 

It was initially proposed that in the absence of p36, the p11 protein was rapidly ubiquitylated on lysines in the carboxyl-terminal region of p11, consequently directing it to the proteasome for degradation [174]. In the study by He et al., overexpression of a series of carboxyl-terminal mutants of p11 and ubiquitin in HEK293 cells showed that ubiquitylation was likely to involve Lys92 or Lys94 of the p11 carboxyl-terminal sequence ^89^VHMKQKGKK^97^. In these experiments, cellular proteins were immunoprecipitated using ubiquitin antibodies and immunoblotted for p11 to determine whether p11 was ubiquitinated. However, it is possible that the proteins immunoprecipitated by the ubiquitin antibodies were ubiquitylated proteins that had bound to p11. Additionally, ubiquitin conjugation of p11 was not confirmed using mass spectrometry. In contrast to the model of ubiquitylation of p11 on carboxy-terminal lysines presented by He et al., Wagner et al. [177] identified Lys47, Lys54, and Lys57 as the potential ubiquitylated lysines in p11 by mass spectrometry analysis of endogenous proteins isolated from murine tissues. However, the study did not address the proportion of p11 of the total population that was ubiquitylated and why ubiquitylated p11 was detected in only two of the five tissues examined. Furthermore, the proposed ubiquitylation sites of p11 were not confirmed by site-directed mutagenesis. Consistent with the findings by Wagner et al., Holloway et al. [178] applied mass spectrometric analysis of overexpressed p11 and identified Lys57 as the primary target of ubiquitylation. This observation was confirmed by site-directed mutagenesis and it was shown that mutagenesis of Lys57 prevented p11 ubiquitylation. Importantly, ubiquitylation of p11 was only observed after overexpression of both p11 and ubiquitin. As the overexpression of proteins can produce cellular anomalies that may induce anomalous ubiquitylation [179], it is unclear if ubiquitylation of p11 occurs under physiological conditions. The formation of ubiquitin chains on the lysine residues of proteins is considered a signal for proteasomal degradation. To form a ubiquitin chain, ubiquitin moieties can be conjugated through one of their lysine residues (K6, K11, K27, K29, K33, K48, and K63) although K29 and K48 are thought to be particularly important. He et al. co-expressed p11 with mutant ubiquitin in which Lys29 or Lys48 of ubiquitin were mutated to arginine (ub-K29R and ub-K48R) in HEK293 cells. They observed that the overexpression of p11 and the ubiquitin mutants increased p11 protein levels, presumably by blocking proteasomal degradation. However, the effect of the mutant ubiquitins was not compared to wild-type ubiquitin to determine if wild-type ubiquitin also modulated p11 expression. Using both the wild-type and a mutant ubiquitin with all lysines mutated to arginine (ub-K0) to prevent any chain formation and any possible proteasomal targeting, Holloway et al. found p11 expression increased in HEK293 cells transfected with both p11 and ubiquitin-wild type (WT) compared to p11 alone. Surprisingly, p11 expression was more dramatically increased when overexpressed with ub-K0, therefore suggesting that ubiquitin chain formation and ubiquitin-dependent proteasomal degradation were not responsible for p11 degradation. Higher molecular weight forms of p11 were detected only when p11 was overexpressed with ubiquitin, and interestingly, multiple molecular weight species of p11 were observed in cells transfected with ub-K0, suggesting the existence of multiple ubiquitin sites in p11 when both proteins were overexpressed. Holloway concluded that the reported ubiquitin-dependent proteasomal degradation of p11 was likely due to an artifact of protein overexpression.

Holloway et al. identified the mechanism of regulation of p11 levels [178]. Using all-trans-retinoic acid (ATRA)-treated acute promyelocytic leukemia (APL) cells, they reported that inhibition of the proteasome with lactacystin prevented the ATRA-induced loss of p11, but the ubiquitylation inhibitor PYR-41 had no effect, suggesting that p11 levels were regulated by a ubiquitin-independent proteasomal mechanism. Furthermore, when proteasomal degradation was inhibited, higher molecular weight ubiquitin-p11 complexes were not detected, although the accumulation of other ubiquitylated proteins was observed. Moreover, incubation of purified p11, p36, and AIIt proteins with the 20S proteasome resulted in the degradation of these proteins In Vitro. Since the 20S proteasome degrades proteins in a ubiquitin-independent manner [180], the authors further concluded that p11 was regulated by a ubiquitin-independent mechanism (Figure 2). In contrast, when p11 and ubiquitin were overexpressed in HEK293T cells, ubiquinylated p11 species were observed. Mass spectrometry analysis of these ubiquinylated p11 species identified lysines 27, 37, and 57 of p11 as sites of ubiquinylation, whereas lysine 57 was the primary site of ubiquitylation, as reported earlier by Wagner et al. [177].

APL is characterized by expression of the PML/RARα, which blocks granulocyte differentiation and has been shown to increase p11 and p36 expression. Treatment of NB4 cells with ATRA, which binds to and activates the destruction of PML/RARα also causes a rapid loss in the expression of p11 and p36 protein. Our laboratory performed an extensive analysis of the mechanism of ATRA-dependent loss of p11 and p36 expression using wild-type NB4 cells, a mutant NB4 cell line resistant to ATRA-induced differentiation (NB4-MR), PR9 cells in which the PML/RARα oncoprotein can be induced by Zn^2+^ treatment, and PML/RARα-negative cells (HL-60, U937, and MCF-7) [178]. We observed that ATRA treatment resulted in the loss of p11 total protein levels in parental and ATRA-induced differentiation-resistant NB4 cells, demonstrating that ATRA-mediated promyelocyte differentiation did not affect p11 or p36 expression. ATRA treatment also diminished p11 and p36 levels in PR9 cells in which PML/RARα was induced and unexpectedly in MCF-7 breast cancer cells, indicating that ATRA affected p11 expression in MCF-7 cells independently of the PML/RARα fusion protein. This suggested that the receptor of ATRA, RARα is involved in regulating p11 transcription. In silico analysis also revealed several potential binding sites for RARα were present in the ±10 kb region from the p11 transcriptional start-site. In addition to this finding, previous studies have reported that ATRA treatment of BEAS-2B lung epithelial cells [181] and dendritic cells [182] also resulted in the downregulation of p11 expression. Lokman et al. found that ATRA treatment resulted in downregulation of p11 and p36 mRNA and cell surface protein levels with a concomitant reduction in plasmin activity, and invasion, in the serous ovarian cancer cell line (OAW28) [183]. Interestingly, a proteomic analysis of tamoxifen-resistant MCF-7-derived cell line with elevated expression of RARα (Lewis lung carcinoma cells [LLC] 2) showed that p11 and several other members of the S100 proteins were reduced compared to parental MCF-7 cells [184]. Considering the presence of potential RARα binding sites in the p11 promoter and that ATRA can modulate p11 expression in the absence of PML/RARα, the use of ATRA may have a therapeutic value in the depletion of p11 and plasmin activity in cancers other than APL. Consistently, the potential of ATRA as a promising new therapy for serous ovarian cancer has been explored by the Ricciardelli group, particularly its effect on regulation of p11 and p36. Their findings suggest that ATRA predominantly downregulated p11 mRNA and protein expression in serous ovarian cancer cells, which resulted in decreased plasminogen activation and invasion in these cells [183]. 

Carnitine palmitoyltransferase 1A has been shown to mediate the succinylation of p11 [185] and that the succinylated form of p11 was upregulated in gastric cancer. The authors showed that p11 was succinylated at lysine residue 47 (K47), which stabilized the protein against proteosomal degradation. Moreover, overexpression of a succinylation mimetic mutant K47E p11 increased the invasion and migration of gastric cancer cells. Interestingly, inhibition of ubiquitination enhanced succinylation of p11. Further studies would be of interest to determine if succinylation modulates the interaction of p11 with p36, plasminogen, or tPA, thus affecting the plasminogen activation functionality. 

Another potential unexplored mode of post-translation modification and regulation of p11 could be via arginylation. Protein arginylation is a post-translation protein modification that is mediated by arginyl-tRNA-protein transferase (ATE1) via the transfer of arginine from t-RNA to N-terminal region of aspartic acid, glutamic acid, or cysteines in proteins [186]. Studies using global arginylation profiling in ATE1 KO mouse models have identified p11 as one of the targets, which was arginylated. The site of arginylation was identified as Ser3 [187]. The physiological significance of arginylation of p11 is yet to be identified, but it would be of interest to determine if arginylation of p11 affects the interaction with its binding partner p36 or other proteins or its localization to the cell surface, stability, and plasminogen receptor function on the cell surface. 

Epithelial-mesenchymal-transition (EMT) is a well-regulated process defined by the gradual loss of epithelial characteristics of the cell and the gain of mesenchymal properties. EMT is crucial not only for embryogenesis and tissue repair but also during cancer progression and dissemination [188,189]. During EMT, the cancer cell acquires enhanced migratory and invasive properties, which include increased expression and production of ECM degrading enzymes such as MMPs [190]. This ultimately leads to cancer metastasis and colonization in distant secondary organs, such as the lungs, bone, brain, and liver, depending on the cancer type. It has been well documented that activation of the transforming growth factor-beta 1 (TGFβ1) signaling pathway orchestrates downstream signaling events and transcriptional programming for the EMT process [191]. Although it has been established that EMT is coupled with increased activity of proteolytic enzymes as the MMPs [192,193,194], the role of the plasminogen activation system has been poorly understood. We have recently shown that p11 is regulated through canonical Smad4-dependent TGFβ1 signaling and repressed by FOXC2-mediated PI3K-mTOR signaling in cells undergoing EMT. Specifically, the components of the plasminogen activation system, namely p11, PAI-1, and uPAR, were differentially regulated during EMT. Interestingly, p11 was the only known plasminogen receptor that was regulated in the A549, a lung cell model, in response to TGFβ1 signaling [195]. This result is further supported by proteomic data by other groups that showed that p11 was one of the few proteins upregulated in mesenchymal prostate cancer cells and A549 cells after TGFβ1 treatment [196,197]. Our studies showed that although total cellular p11 expression was increased in response to TGFβ treatment, plasmin generation or invasiveness was not enhanced (Figure 2). This was primarily due to increased PAI-1 expression and reduced cell surface expression of p11 [195]. Other studies have observed that both uPAR signaling and PAI-1 expression are required for activation of EMT in breast cancer [198] and fibroblasts [199], respectively. These studies have suggested that p11 is one of the crucial plasminogen receptors for EMT and that cell surface expression of p11 drives the plasminogen activation during EMT. Although paradoxical that PAI-1, a major plasminogen activation inhibitor, is upregulated during EMT, it is potentially necessary to maintain the delicate balance between plasminogen activation and inhibition during the invasive escape of cancer cells. 

## 9. Role of p11 in Plasmin Generation

The AIIt heterotetramer is a co-receptor for both plasminogen and tPA. The p36 subunit serves to anchor the complex to the cell surface while the p11 subunit serves as a plasminogen receptor. Plasminogen was initially shown to bind to the carboxy-terminal lysine of the p11 subunit [162,172]. p11 has a high affinity for tPA (Kd of 0.68 µM) and plasminogen (Kd of 0.11 µM). As with other plasminogen receptors, plasminogen binding to p11 induces an open conformation of plasminogen that promotes its activation [100,162]. Since the cleavage of the carboxyl-terminal lysines of p11 by carboxy peptidase B or their removal by site-directed mutagenesis results in a loss in plasminogen binding and activation, it has been assumed that the carboxy-terminal lysine of p11 is the key regulators of plasminogen binding and activation. However, our laboratory recently showed that the substitution of the carboxyl-terminal lysines with isoleucine (p11K95,96I) or arginine (p11K95,96R) did not result in a loss in p11-dependent plasminogen activation [100]. Furthermore, we reported that ε-aminocaproic acid, a lysine analog that blocks the lysine-binding kringle domains of plasminogen, blocked acceleration of plasminogen activation by the p11K95,96I mutant. Hence, these findings suggested that p11 uses internal lysine residues to bind plasminogen and tPA and that loss of the last two carboxyl-terminal residues of p11 may result in destabilizing p11 such that the internal lysine residue is not available for plasminogen activation. 

In the same study, we also investigated the structural interactions between p11, tPA, and plasminogen. We observed that in contrast to the reported critical function of the finger domain of tPA in fibrin-stimulated plasmin generation, the kringle-2 domain of tPA played a key role in p11-dependent plasmin generation. We then showed that the kringle-2 domain of tPA was critical for plasminogen activation by other plasminogen receptors including, enolase-1, S100A4, histone H2B, cytokeratin 8, and high mobility group box-1 protein. Next, we used a series of plasminogen kringle mutants to identify the domain of plasminogen key for p11-dependent plasminogen activation. The kringle-1 domain of plasminogen, previously shown to be indispensable for fibrin-binding was shown to also be critical for p11-dependent plasmin generation. 

The depletion of p11 via shRNA has been shown to dramatically reduce plasmin generation in a variety of cell types. For example, the depletion of p11 in HT1080 fibrosarcoma cells reduced plasmin generation by up to 95%, and ECM hydrolysis was reduced by 70% compared to HT1080 control cells [200]. AIIt purified from bovine lung was initially reported to dramatically stimulate plasmin generation; yet, purified annexin proteins, including p36, were shown to have a marginal effect on plasmin generation [160]. Later, Kassam et al. [75] found that plasmin generation by the AIIt heterotetramer stimulated activation of glu-plasminogen by 341-fold, whereas the p36 monomer stimulated activation by 6-fold. They also investigated the role of p11 in plasmin generation by comparing human recombinant proteins. They found that tPA-dependent plasmin generation was stimulated 77-fold by AIIt that was formed by the combination of recombinant p11 and p36, 46-fold by p11, and only 2-fold by p36. They also observed that the combination of p11 with the first 15 amino acids of the p36-amino-terminus, which contained the p11-binding sites, was like AIIt formed with the full-length p36. Hajjar’s group has reported that Cys-8 in the amino-terminal domain of p36 forms the binding site for tPA and binds tPA with a Kd of 48 nM [201]. They also reported that the Cys-8 residue of p36 is a key target for homocysteinylation and that this residue forms a disulfide bond with homocysteine. Importantly, the homocysteinylation of this site, reported by this group, was shown to block plasmin generation by p36 In Vitro and to account for the hemostatic imbalance displayed by patients with hyperhomocysteinemia. However, we reported that the AIIt composed of p36 (Cys-8-Ser)/p11 wild-type (WT) (AIIt Cys8Ser) exhibited greater acceleration of tPA-dependent plasminogen activation than the wild-type AIIt. This rules out the postulated role of the Cys-8 residue of p36 in plasminogen activation. Hence, our data firmly support the concept that the p11 subunit and not p36 is the critical regulator of plasmin generation by the AIIt heterotetramer. As part of AIIt, the p11 subunit has a key function in the binding of plasminogen and facilitating its activation by binding and co-localizing plasminogen activators, yet p11 is also capable of binding newly generated plasmin to protect it from inactivation by α_2_-anti-plasmin [75]. 

Kwon et al. [78] also found that plasmin binding to p11 could promote plasmin-autoproteolysis. The specific plasminogen fragments produced from plasmin autoproteolysis are called angiostatins. These are a group of angiogenesis inhibitors. Kwon demonstrated that cells convert plasminogen to a form of angiostatin called A61 (Lys78–Lys468) in a three-step process. Initially, uPA cleaves the Arg561-Val562 of plasminogen which results in the formation of plasmin. Next, plasmin autoproteolysis results in the cleavage of the Lys77–Lys78 and Lys468–Gly469 bond. However, the presence of a Cys462–Cys541 disulfide prevents release of A61 (Lys78–Lys468) from the plasmin molecule. Last, AIIt catalyzes the reduction of the Cys462–Cys541 disulfide of plasmin, which allows the release of A61 from plasmin. Mutagenesis of p36 Cys334Ser and either p11 Cys61Ser or p11 Cys82Ser was observed to inactivate the plasmin reductase activity of the isolated subunits, which suggested that specific cysteine residues of both p36 and p11 participated in the plasmin reductase activity of AIIt.

## 10. Involvement of p11 in Biological Processes and Cancer

As described above, since AIIt heterotetramer serves as a scaffold protein for protein–protein interactions with several other proteins, p11 plays a crucial role in several other biological processes via the virtue of this interaction. These processes include Na^+^ absorption, pain processing, neuroprotective role in amyotrophic lateral sclerosis and traumatic model of motor neuron degeneration, and finally depression. The role of p11 in these processes has been discussed extensively elsewhere and will not be the focus of this review [161]. 

Plasmin generation by the p11 subunit of AIIt is involved in multiple biological processes including monocyte and macrophage recruitment in inflammatory responses [202,203], fibrinolysis by endothelial cells [204], and cancer cell metastasis [167,200,205]. The presence of p11 at the cell surface of endothelial cells plays a vital role in plasmin generation that participates in vascular fibrinolysis. The shRNA-depletion of p11 from telomerase immortalized microvascular endothelial cells resulted in the dramatic decrease of plasminogen binding and plasmin generation by 50% and 60%, respectively, without affecting the presence of p36 at the cell surface [204]. Interestingly, the depletion of p36 reduced plasminogen binding and plasmin generation to a similar extent in these cells. As p36 has been shown to play a vital role in the stabilization and localization of p11, the findings indicate p11 is essential for the plasmin generation in endothelial cells, whereas p36 plays a role in the stabilization of p11. Surette et al. [204] also found that endothelial cell p11 was required for plasmin-dependent fibrinolysis In Vivo. In p11-KO mice, fibrin deposition was increased compared to p11-WT mice, and this was found to be independent of coagulation since there was no difference in prothrombin time and activated partial thromboplastin time between p11-WT and p11-KO mice. Using a tail-clip experiment to characterize the length of time for bleeding to stop, the time to stop bleeding for p11-KO mice was 4-fold less than the p11-WT group, thus indicating that the loss of p11 reduced plasmin-dependent fibrinolytic activity. 

Cell surface plasmin generation via p11 plays a major role in the migration of macrophages to the site of inflammation [203] and tumor sites [202]. The plasmin generated at the cell surface of macrophages facilitates their migration through tissues by direct hydrolysis of the protein that forms the ECM and by activating MMPs [206] that will further degrade the ECM upon activation. Other studies have shown that p11 plays a crucial role in sepsis in animal models by negatively regulating macrophage Toll-Like-Receptor (TLR) signaling [207]. 

The aberrant expression of p11 has been found to promote the invasiveness of tumor cells and metastasis by numerous studies. In mouse xenograft models, transplantation of p11-depleted HT1080 fibrosarcoma cells showed a 3-fold reduction in the number of metastatic foci in the lungs compared to mice injected with HT1080 cells with WT levels of p11 [200]. In contrast, the overexpression of p11 in the HT1080 fibrosarcoma cells resulted in a 16-fold increase in the number of metastatic foci in the lungs [200]. Depletion of p11 in colorectal cell line CCL-222 reduced plasminogen binding by 45% and plasmin generation by 65%, and these cells were unable to migrate through a Matrigel barrier in a plasmin-dependent manner [205]. Recently, Bydoun et al. demonstrated that loss of p11 in a pancreatic cancer cell line (Panc1) resulted in decreased plasminogen activation and plasminogen-dependent invasion with no alteration in tumor cell proliferation In Vitro. Interestingly, the injection of the p11 depleted pancreatic tumor cells in immunocompromised non-obese diabetic (NOD)/severe combined immunodeficiency (SCID) mice resulted in reduced tumor growth compared to control cells, suggesting the involvement and interaction with TME in tumor growth [208]. Many studies in other cancer models have also associated p11 in tumorigenesis through plasminogen-independent mechanisms. For example, loss of p11 in ovarian cancer cells resulted in reduced tumor growth in NOD/SCID mice In Vivo with concurrent reduction of cell proliferation, colony formation, migration, and invasion In Vitro. Moreover, p11-depleted cells show decreased resistance to carboplatin In Vitro [209]. Li Y et al., recently demonstrated the importance of p11 in gastric cancer tumorigenesis. Overexpression of p11 in gastric cancer cells resulted in increased tumor burden in subcutaneous tumors in BALB/c-nude mice. Rapamycin treatment of the subcutaneous tumors from p11 overexpressing cells resulted in a significant reduction in tumor weight and size suggesting that p11 promotes tumor growth in gastric cancer cells via mTOR signaling pathway. Consistently, these authors also observed a positive correlation between p11 expression and mTOR signaling in Gene Set Enrichment Analysis from The Cancer Genome Atlas database. More specifically, these studies have indicated that p11 stimulates aerobic glycolysis in gastric cancer cells via activation of Src/ANXA2/AKT/mTOR signaling pathway, ultimately leading to enhanced cell proliferation and resistance to apoptosis. This is the first investigation directly implicating p11 in aerobic glycolysis [210]. Two recent studies have highlighted the importance of p11 in modulating gene expression of pluripotency factors in breast cancer stem cells (CSC). Studies by Yanagi et al. have shown that p11 was dramatically upregulated in CD44+ metastasized CSC in mouse patient-derived xenotransplant (PDX) mammary tumors compared to CD44− metastasized and CD44+ and CD44− primary CSC. Moreover, overexpression of p11 in human breast cancer cell line MDA MB 231 resulted in increased organoid-forming, invasive, and metastatic capacity, whereas loss of p11 resulted in a reduced number of organoids. Interestingly, these studies also established that p11 upregulated stem cell-related genes, such as OCT4, SOX2, and NANOG, in the organoids, suggesting a novel function of p11 in promoting metastasis of CSC [211]. Lu et al., observed that chemotherapy (paclitaxel or carboplatin)-induced p11 is mediated by HIF-1, which further promotes epigenetic modulation and transcriptional activation and expression of pluripotency factors, such as NANOG, SOX2 and KLF4, in breast cancer cell lines. Moreover, loss of p11 resulted in increased sensitivity to paclitaxel in orthotopic mammary tumors [212]. Both these studies emphasize the pivotal role of p11 in facilitating the enrichment of CSC in breast cancer. Although these authors did not investigate the plasminogen activation function of p11 in these cell models, it is highly likely that p11 plays both plasminogen-dependent and -independent function in tumor cell proliferation, migration, and invasion. Identification of the lysine residue involved in plasminogen binding and generation of p11 mutant mice incapable to activating plasminogen will further our understanding of plasminogen-dependent and -independent functions of p11 in cancer progression.

## 11. Plasmin Functions in the Tumor Microenvironment—Regulation of Macrophage Function

In the past two decades, the major focus on the plasminogen activation system has been on the various components specifically the activators, inhibitors, and receptors. However, the effects of the proteolytic plasmin on cancer progression have been poorly understood. Plasmin can cleave a broad range of ECM substrates, growth factors, and cytokines. The plasmin-mediated function in TME can be classified into two categories—proteolytic processing of substrates and proteins and plasmin signaling at the cell surface. 

Plasmin has been indirectly implicated in the development and progression of tumors by activating various growth factors, such as TGFβ, FGF-2, and HGF. TGFβ and uPA are coordinately and tightly regulated in cancer progression. TGFβ upregulates uPA expression, which further enhances plasmin generation and activates TGFβ which contributes to the tumor progression via its effects on EMT, invasion, and metastasis (reviewed in [213,214]). 

As discussed, several components of the plasminogen-plasmin system including activators, inhibitors, and receptors, are expressed on stromal cells and concurrently support tumor growth and progression. For instance, uPA is expressed on myofibroblasts and uPAR; plasminogen receptors, such as Plg-RKT and p11, are expressed on the macrophages; and PAI-1 on macrophages, endothelial cells, and fibroblasts. Studies using transgenic and ectopic mouse tumor models have played a tremendous role in our understanding of the stromal and cancer cell contribution of the plasminogen -plasmin system components toward cancer progression. 

The role of the proinflammatory function of plasmin during tissue injury and atherosclerosis is well known. Studies have demonstrated that binding of plasmin to monocytes induces expression of proinflammatory cytokines, such TNF-α and IL-6, via the activation of NF-κB and JAK/STAT pathway [215]. AIIt is required not only for plasmin binding on monocytes but also for plasmin-mediated downstream signaling. This function of plasmin is independent of the catalytic/proteolytic function of plasmin [216].

The generation of plasmin from plasminogen by tPA has been shown to regulate the balance of soluble and cell-bound levels of the chemokine CCL21 in the immune system, particularly in the dendritic and T-cells. This chemokine has a crucial role in the migration and homing of T-lymphocytes and antigen-presenting dendritic cells [217]. In another study, plasmin stimulated chemotaxis of monocyte-derived dendritic cells via the activation of Akt2 and p38 mitogen-activated signaling pathway [218]. These studies suggest that plasmin is a potent chemoattractant for immune cells acting via its proteolytic and signaling function. Although studies for the role of plasmin signaling have not been performed in the context of tumor growth, progression, and TME, it is well established that tumor-infiltrating immune cells play an important role by either interfering or promoting tumor progression [219]. 

In the context of cancer, studies have shown the dual requirement of the proteolytic and signaling properties of plasmin for promoting a motogenic phenotype. For example, in HT29-M6 intestinal cell lines, treatment with phorbol 12-myristate 13-acetate (PMA) resulted in a cascade of events, which involved initial upregulation of uPA, proteolysis of uPAR, activation of plasminogen to plasmin degradation of ECM, and scattering of cells. Activation of ERK1/2 was required for the cell scattering event that was mediated by plasmin intracellular signaling [93]. 

The plasminogen-activation system plays a pivotal role in macrophage biology, specifically macrophage migration and phagocytosis. In one of the first studies, Ploplis et al. used plasminogen-deficient mice and demonstrated that upon inflammatory stimulus with thioglycolate, monocyte and lymphocyte recruitment to the peritoneal cavity was dramatically retarded [220]. More recently, Silva et al. demonstrated that the decrease in macrophage recruitment could be partly attributed to a decrease in plasmin-mediated fibrinolysis in these mice. In this study, they showed that reduced peritoneal infiltration of macrophages in plasminogen-depleted mice was restored by fibrinogen deficiency [221]. Further evidence for the signaling role of plasmin in mediating monocyte migration was obtained from studies by Carmo et al. These studies have shown that plasmin facilitates monocyte migration via PAR-1, MEK/ERK, and CCL2/CCR2-mediated signaling [90]. Das et al. showed a remarkable decrease in macrophage-mediated phagocytosis of apoptotic thymocytes in spleen and reduced uptake by peritoneal macrophages in plasminogen knockout mice. Overall, the spleen and liver from these plasminogen-deficient mice displayed downregulation of genes involved in phagocytosis [222]. 

**Figure 3:**
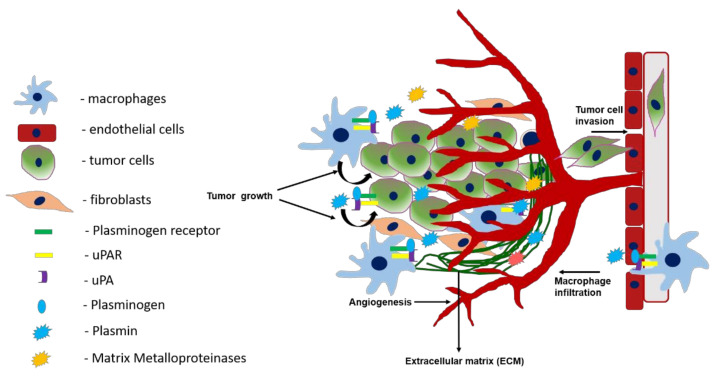
Role of the plasminogen activation system in tumor growth, angiogenesis, macrophage infiltration, and invasion. Activation of plasminogen bound to plasminogen receptors at the cell surface of macrophages and tumor cells is mediated by uPA bound to uPAR. This results in the production of plasmin which activates matrix metalloproteinases and together these proteases play a key role in the infiltration of macrophages to tumor sites. At the tumor site, macrophages promote tumor growth, angiogenesis, and metastasis. Plasmin produced by the tumor cells mediates tumor cell migration and invasion both by protease dependent and independent mechanisms.

Aligned with the role of plasminogen in macrophage recruitment (summarized in Figure 3), depletion of several plasminogen receptors results in a phenotype resembling that in plasminogen-deficient mice. The role of plasminogen receptors on monocyte migration was mostly studied using an inflammation model. Receptors, such as enolase-1, histone H2B, Plg-RKT, and p11, played predominant roles in thioglycolate elicited monocyte migration both In Vivo and In Vitro. Enolase-1 was shown to play a crucial role in lipopolysaccharide (LPS)-driven monocyte migration and matrix degradation In Vitro and LPS-dependent recruitment of alveolar monocytes In Vivo. LPS upregulated enolase-1 in human blood monocytes and U937 cells, which in turn resulted in plasminogen-dependent enhancement in monocyte recruitment [223]. Das et al., showed that although multiple plasminogen receptors, such as enolase-1, AIIt, and histone H2B, are expressed in the macrophage cell lines RAW 264 and JA774.1 cells, histone H2B contributed to 50% of the plasminogen activation function in these cells [147]. The newest member of the plasminogen receptor family, Plg-RKT, is the only transmembrane plasminogen receptor. This receptor is expressed in blood monocytes and monocytoid cells. Inhibition of Plg-RKT by blocking antibodies resulted in a dramatic reduction in plasmin generation, monocyte migration, and invasion through a Matrigel-coated chamber. Moreover, in the thioglycolate dependent peritonitis model, treatment with Plg-RKT antibody also resulted in the reduction of macrophage recruitment in the peritoneal cavity [224,225]. Our laboratory has shown the role of p11 in plasmin-dependent macrophage infiltration in both inflammation and tumor models. The availability of p11-KO mouse models has enabled an elegant demonstration of the important contribution of p11 toward macrophage recruitment. O’Connell et al. [203] examined how the loss of p11 affected the migration of macrophages using the thioglycollate-induced peritonitis as a model of inflammation. In p11-KO mice, the number of macrophages that migrated into the peritoneal cavity upon thioglycolate treatment was reduced by 53% compared to the macrophages of p11-WT mice. Furthermore, the p11-KO macrophages displayed limited invasive capability using the Matrigel plug assay In Vivo. 

Phipps et al. [202] discovered that the expression of p11 in macrophages plays an essential role in facilitating macrophage migration to tumors. Using a xenograft mouse model in which LLC cells were transplanted into p11-WT and p11-KO mice, tumor growth was arrested after seven days in p11-KO mice. In contrast, tumor growth continued in the p11-WT mice, where tumors were 10-fold larger than those in p11-KO mice. In p11-WT mice, the immunohistochemical analysis determined that macrophages were present throughout the tumor. In contrast, macrophages were present at the periphery of tumors in p11-KO mice, indicating that macrophages lacking p11 were incapable of infiltrating into tumors. To determine the impact that p11-expression in macrophages has in affecting tumor growth in the p11-KO mice, p11-WT macrophages were injected peritoneally into p11-KO mice before transplantation of LLC cells. Tumors in the p11-KO mice transplanted with p11-WT macrophages presented tumors comparable to the tumors observed in p11-WT mice, hence indicating that p11-dependent plasmin generation in macrophages plays a critical role in facilitating tumorigenesis.

Currently, research on the role of p11 in tumorigenesis has been predominately conducted in ectopic tumor models. Transgenic mouse models of cancer are histologically and genetically accurate models of human cancer. Recently, we utilized the mouse mammary tumor virus-polyoma middle tumor (MMTV-PyMT) transgenic model to interrogate the role of p11 in breast cancer in WT and p11-KO mice [226]. The MMTV-PyMT transgenic model-specific expression of the oncogene PyMT under the MMTV promoter, results in widespread tumor growth in the mammary glands and spontaneous metastasis to the lymph nodes and lungs. This mouse model is very similar to human breast cancer in that the tumors display histological and molecular characteristics mirroring the progression of human breast cancer. With this model system, we have shown that p11 is exclusively expressed in the stromal compartment, and the loss of p11 dramatically reduces tumor onset, growth, and progression. Spontaneous and experimental pulmonary metastasis were also substantially reduced in the absence of p11. Surprisingly, tumors and tumor cell lines isolated from these animals do not demonstrate a decrease in plasminogen activation with the loss of p11. We hypothesize that plasmin generation by stromal cells, such as macrophages, is critical for tumor growth and progression of breast cancer in this model system. Many dysregulated pathways and processes observed in breast cancer progression mimic those observed during normal mammary gland development and tissue remodeling. During puberty, and under the control of hormones and other factors, the ductal epithelium of the rudimentary mammary gland invades into the mammary fat pad in a process referred to as branching morphogenesis. ECM-degrading enzymes, including MMPs and their inhibitors, tissue inhibitors of metalloproteinases are involved in this process [227]. Plasminogen-deficient mice show a delay in early ductal development and many are unable to lactate because of a lack of secretory epithelium [228]. Since p11 is involved in cancer cell invasion we investigated the possibility that p11 might also be involved in branching morphogenesis. We observed a reduction in length of the ductal branches in the p11-KO mammary glands at the early time points of 6 and 8 weeks and was comparable to the WT at 10 and 12 weeks. This suggests that p11 regulates the invasion of both normal and cancer cells.

Hence, there is ample evidence linking the plasminogen activation system components to macrophage invasion and migration (Figure 3). Several studies have demonstrated that plasminogen plays an important role in macrophage-driven phagocytic function in the context of liver injury and wound healing. Using plasminogen and uPA KO mice, Kawao et al. showed that in a liver injury model, plasminogen was important for macrophage-specific phagocytosis of cellular debris [229]. On the contrary, Rosenwald et al. showed that plasminogen does not induce macrophage-specific phagocytosis but rather affects the prey cells [230]. Finally, Das et al. showed using plasminogen-deficient mice that many genes involved in phagocytosis are downregulated in these mice and that plasminogen is a key regulator of macrophage-specific phagocytosis [222]. Although there is no direct evidence of the role of plasminogen and phagocytosis in the context of tumor and TME, this might be mainly relevant in chemotherapy and radiation therapy. In this scenario, potentially damage associated molecular patterns induced by dying tumor cells can activate endothelial cells and promote migration of myeloid cells, such as macrophages, which in turn results in the clearance of apoptotic tumor cells by phagocytosis. Future studies should be aimed at identifying the plasminogen receptors responsible for the phagocytosis process.

Initially, the role of the uPA, uPAR, plasminogen, PAI-1, and plasminogen receptors in cancer growth was studied using transplantable and ectopic tumor models in NOD/SCID and syngeneic mice and antibody-targeting and anti-sense gene knock-down technology. These studies primarily utilized LLC cells, melanoma, squamous lung carcinoma, and prostate cancer models [105,231,232,233,234,235,236,237]. Overall, these studies showed that inhibition of uPA and uPAR resulted in decreased metastasis. More recently, due to the availability of gene knockout transgenic mouse models, the role of plasminogen, uPA, uPAR, PAI-1, and plasminogen receptors in tumor growth and metastasis have been extensively studied. The MMTV-PyMT transgenic model has been extensively used to study the function and expression of the various components of the plasminogen activation system (reviewed in [238]). More importantly, this model is relevant to understanding the expression and contribution of the plasminogen activation components by both TME and tumor cells in cancer growth and progression. In situ hybridization and immunohistochemistry have revealed that the expression of uPA, uPAR, and PAI-1 is predominantly increased in the stromal compartment, by cells that include fibroblasts, macrophages, and endothelial cells. Interestingly, this mirrors the expression and localization pattern in human breast cancer, where the highest uPA [239] and uPAR [240] expression is found in stromal cells including the macrophages in the periphery of breast cancer cells, whereas PAI-1 expression is found predominantly in the myofibroblasts [241]. Studies with plasminogen-deficient mice that express the MMTV-PyMT oncogene presented contrasting results among different groups. The first studies performed in the late ’90s by the Bugge group showed that loss of plasminogen decreased spontaneous pulmonary metastasis with no effect on tumor onset and growth [53]. In contrast, studies reported by the Lundt group more than a decade later showed that loss of plasminogen did not decrease spontaneous metastasis to the lung. The latter group attributed this confounding result to the extensive backcrossing of their mice before initiating these studies [242]. In the MMTV-PyMT model, there is no significant difference in tumor growth and vascularization between WT and uPA, uPAR, and PAI-1 deficient animals [238]. The uPA deficiency shows the most significant decrease in metastasis, and PAI-1 depletion has a slight metastasis promoting effect (although not statistically significant); uPAR loss does not affect metastasis (reviewed in [238]). Interestingly, the effect of uPA on cancer progression depends on the organ and cancer types. In the case of melanoma, uPA deficiency in mice results in decreased invasion and progression, whereas inhibition of uPA does not affect the progression of pancreatic cancer in the RIP-Tag2 transgenic mouse model [243,244]. Among the PgRs, genetic depletion of S100A4 resulted in a dramatic decrease in pulmonary metastatic burden without any alteration in tumor onset and growth kinetics. This was accompanied by significant suppression of T-cell infiltration in the tumors depleted of S100A4. S100A4 secretion in the TME resulted in the influx of T-cells which release cytokines resulting in enhanced metastasis [245]. Our studies with the MMTV-PyMT transgenic model in which the p11 gene was knocked out was the only instance of a plasminogen activation system component (p11) playing a dynamic role in tumor onset, growth, progression, and metastasis. 

## 12. Plasminogen Activation Components and Role in Chemoresistance 

There are two main molecular targets in breast cancer based on the expression of estrogen receptor alpha (ERα) and epidermal growth factor receptor 2 (HER2). Tamoxifen is the main component for the treatment of ERα positive breast cancer, which accounts for about 70% of all breast cancers. Tamoxifen acts by competing with estrogen for its binding with ERα resulting in the inhibition of estrogen effect on cancer cell proliferation (reviewed in [246,247]). Although tamoxifen therapy has significantly resulted in reduction in mortality rate in ER-positive breast cancer patients, a significant proportion of patients present with recurrence due to intrinsic or acquired resistance to tamoxifen. Components of the plasminogen activation system have been involved in predicting tamoxifen resistance in hormone responsive breast cancer. A high tumor expression of uPA, uPAR, and PAI-1 was associated with poor response to tamoxifen in patients with recurrent breast cancer [248,249]. 

Chemotherapy is the primary mode of treatment for metastatic and aggressive breast cancer especially triple-negative breast cancer. Triple-negative breast cancer is characterized by loss of ER, progesterone receptor and HER2 and is highly aggressive and metastatic. Chemotherapeutic agents, such as paclitaxel and carboplatin, are commonly used to treat aggressive breast cancer. Although these drugs reduce overall primary tumor burden, many patients develop recurrent and metastatic disease which are subsequently resistant to these drugs. Hence, chemoresistance remains a major barrier to successful treatment of aggressive and metastatic breast cancer. Several mechanisms contribute toward chemoresistance that is well studied specifically for breast cancer. These include but are not restricted to increased drug metabolism and excretion of the drugs, mechanisms for resisting apoptosis and enhancing cell survival pathways, EMT, activation of cancer stem cells, efficient DNA damage response and repair, and finally, alterations in the TME to promote drug resistance (reviewed in [250,251]).

p11 has been linked to resistance to several chemotherapeutic agents. Proteomic analysis of acquired tamoxifen resistance in MCF-7 breast cancer cells has identified p11 as one of the top five upregulated proteins in tamoxifen-resistant cells [252]. Similarly, proteomic analysis showed that p11 was significantly increased in relapsing patients associated with tamoxifen resistance [184]. Overexpression of p11 has also been linked to reduced sensitivity of colorectal cancer cells [253] to oxaliplatin and depletion of p11 in leukemic cells increases sensitivity to vincristine [254]. Finally, p11 was part of an 11-member signature panel associated with multi-drug resistance in ovarian cancer [255]. The functional significance of p11 in promoting chemotherapy resistance has not yet been elucidated, but these studies allude to its potential as a prognostic marker.

## 13. The Role of the Plasmin and Plasminogen System as Biomarkers in Cancers

Cancer biomarkers are most often used as aid in diagnosis but can also be useful to determine tumor aggressiveness. Biomarkers also play a key role in identifying new personalized targets depending on the aggressiveness of the disease and toxicity to the therapeutic agents. In order for personalized therapies to be effective we require validated novel biomarkers for predicting aggressive vs. non-aggressive cancer, response to therapy and therapeutic resistance, and for predicting therapeutic side-effects and toxicity [256]. The best-established cancer biomarkers are uPA/uPAR and PAI-I [42,257]. As discussed, many components of the fibrinolytic system play an important pathologic role in tumor growth and metastases. Ultimately, plasmin plays two distinct roles in the transformation process. First, plasmin participates in the proteolytic activation of several growth factors such as TGFβ, FGF2, ILGF-1, and hepatocyte growth factor. Second, plasmin participates in tumor growth and metastasis via degradation of components of the extracellular matrix which form a barrier to metastasis. Plasmin also participates in the activation of several proteases, such as the MMPs, that also proteolyze extracellular matrix components. The uPA/uPAR complex also plays a role in signal transduction and cellular adhesion. Substantially increased levels of uPA were observed in various cancers of the breast, stomach, colon and rectum, esophagus, pancreas, glioma, lung, kidney, prostate, uterine cervix, ovary, liver and bone as evaluated by tissue extraction, immunohistochemical and in situ hybridization methods [257]. Subsequently, the increased expression of uPA, uPAR, and/or PAI-1 has been demonstrated to be associated with poorer overall prognosis in multiple cancer types, including cancer of breast [258], bone [259], colon and rectum [260,261,262], pancreas [263], glioma [264], kidney [265,266], prostate [267,268], lung [269], cervix [270], ovary [271], esophagus [272], liver [273] and stomach [274].

Of the various cancers, the best-established case for the utilization of uPA/PAI-1 as biomarkers is for breast cancer. The use of uPA/PAI-1 as prognostic biomarkers in lymph node-negative breast cancer has been confirmed by randomized prospective clinical trials involving pooled analysis of data from retrospective and prospective studies [275]. In this regard they are used for the identification of lymph node-negative patients who have HER-2-negative tumors and who can be safely spared the toxicity and costs of adjuvant chemotherapy.

The association of uPA and PAI-1 with tumor aggressiveness and patient response has been supported by an extensive array of studies spanning over 30 years. However, the use of uPA/uPAR and PAI-1 as biomarkers in clinical practice has been very limited. A major reason for this has been the fact that while inhibition of uPA, uPAR or PAI-1 by inhibitory monoclonal antibodies, by gene manipulations such as the transfection of PAI-1 into experimental tumors, and by synthetic small molecules designed to target uPA, uPAR or PAI-1 have shown in many cases dramatic inhibition of tumor growth and metastasis in experimental animals, these approaches have had minimal impact on human tumors and metastases. In the absence of clinical trials showing significant effects on human cancers, there has been a reluctance to use these components as biomarkers. 

The studies on the expression of plasminogen receptors as cancer biomarkers have been performed on a few known plasminogen receptors such as p11, Enolase 1, and cytokeratin 8 [155,156,159]. Many of the plasminogen receptors are multifunctional proteins with both intracellular and extracellular expression and functionality in cancer progression. Hence, it is imperative to specifically delineate the intracellular and membranous (or secreted) expression patterns of the receptors when describing the diagnostic, and prognostic value of the receptors in human cancers. Nevertheless, overexpression of several of the plasminogen receptors has been correlated with poor overall survival and prognosis. 

Clinical analysis has demonstrated that p11 gene and protein expression are upregulated in several cancers, such as blood, brain, colorectal, gallbladder, kidney, lung, lymphatic, ovary, pancreas, prostate, skin, stomach, and thyroid, and it has been extensively documented in recent reviews [156,276,277]. Our laboratory has recently established the potential of p11 as a diagnostic and prognostic biomarker in pancreatic and breast cancer. In silico analysis of pancreatic tumors and cell lines demonstrated upregulation of p11 mRNA expression. p11 mRNA expression and methylation status were predictive of overall survival and recurrence-free survival (RFS) in pancreatic ductal adenocarcinoma patients. Furthermore, protein expression of p11 significantly increased in during progression from normal to pancreatic ductal adenocarcinoma as demonstrated in tissue microarray analysis in human patients [208]. 

Expression of p11 in breast cancer has been analyzed in multiple studies with inconsistent observations. In one of the early studies, Carlsson et al. performed an analysis in ductal carcinoma in situ and invasive and metastatic breast tumors using serial analysis of gene expression. They found that p11 gene expression was downregulated in breast cancer irrespective of pathological grade [278]. In contrast, Yu et al. demonstrated that p11 was one of the 170 genes that were activated at the intravasation step during metastasis [279] in breast cancer patients. Studies by Zhang et al., using Kaplan—Meier plotter database showed that elevated p11 mRNA expression was predictive of worse overall survival (OS) in basal-type breast cancer, and there was no significant association of p11 expression in other subtypes based on clinicopathological grades [280]. Since p11 protein expression is subjected to multiple post-translational modifications and interactions with several binding partners, gene expression analysis alone will not suffice to understand the clinical significance of p11 expression. To date, only two studies have examined the expression of p11 protein in clinical samples from breast cancer patients. The first analysis by Arai et al. demonstrated membranous overexpression of p11 in high-grade tumor status correlating with high mitotic counts, severe nuclear pleomorphism, high Ki67 positivity index and low ER status [281]. They did not observe any correlation of p11 protein expression with stromal invasion. Our laboratory has utilized both gene expression profiling and immunohistochemical staining for p11 in breast cancer patient tissues. We observed that p11 mRNA expression was significantly associated with poor patient prognosis and significantly elevated in high-grade, triple-negative tumors, and tumors with high proliferative index. Most impressively, we observed that the high mRNA levels of p11 were significantly associated with poor patient OS and a hazard ratio of 3.34. Although membranous expression of p11 protein as evaluated by immunohistochemistry was significantly elevated in tumor cells compared to normal mammary epithelium, there was no correlation between high p11 expression and clinical and pathological tumor grade or with molecular subtype in human breast cancer samples. The discrepancy between p11 mRNA and protein expression in human tissues can be partially attributed to the contribution of p11 by stromal cells in the TME, which has not been examined in the immunohistochemistry analysis. Future studies should be focused on employing a larger cohort of tissues for a detailed evaluation of stromal and tumor cell expression of p11 in breast cancer [226].

Similarly, mRNA and protein for cytokeratin 8 is overexpressed in several cancers such as breast, colon, gastric, head and neck, liver, lung, skin and uterus (reviewed in [155]). Enolase 1 has also been evaluated for its use as a biomarker in several cancers, where is shown to be upregulated such as bone, breast, head and neck, liver, lung and pancreas where it suggests to have a prognostic significance (reviewed in [155]. Since many of the plasminogen receptors play a key role in the macrophage function in animal models as described previously it will be interesting to determine if macrophages in human tumors from patients show elevated expression of plasminogen receptors, which can further be used as a prognostic indicator of cancer progression and therapeutic interventions. 

## 14. Concluding Remarks

Overall, the interpretation of the role of the plasminogen activation system in tumor progression has been more complicated than expected due to the multifunctionality of the components and their plasmin-independent role in tumor progression. For instance, plasmin apart from its enzymatic function also promotes cell signaling which potentially contributes toward tumor growth and metastasis. uPAR is a pleiotropic protein that regulates cell adhesion, migration, proliferation, via intracellular signaling and independent of its plasmin activating function.

In summary, from these studies, we can infer that plasmin-mediated proteolysis contributes to the degradation of ECM, which can directly or indirectly affect tumor stroma formation, angiogenesis, and dissemination. Plasmin generation by stromal cells, such as macrophages is critical for tumor growth and progression in many forms of cancer including breast cancer. In this regard, the plasminogen receptors play a key role in regulating plasmin generation. Our work has highlighted the importance of the plasminogen receptor, p11, in the interaction of cancer cells with the stroma.
